# Artificial optoelectronic synapse based on CdSe nanobelt photosensitized MoS_2_ transistor with long retention time for neuromorphic application

**DOI:** 10.1515/nanoph-2024-0368

**Published:** 2024-08-29

**Authors:** Xiaohui Song, Xiaojing Lv, Mengjie He, Fei Mao, Jie Bai, Xuan Qin, Yanjie Hu, Zinan Ma, Zhen Liu, Xueping Li, Chenhai Shen, Yurong Jiang, Xu Zhao, Congxin Xia

**Affiliations:** Henan Key Laboratory of Photovoltaic Materials, Department of Physics, 66519 Henan Normal University, Xinxiang 453007, China; Department of Electronic and Electrical Engineering, 66519 Henan Normal University, Xinxiang 453007, China

**Keywords:** cadmium selenide, molybdenum disulfide, heterojunction, artificial synapse, neuromorphic application

## Abstract

Optoelectronic synaptic devices have been regarded as the key component in constructing neuromorphic computing systems. However, the optoelectronic synapses based on conventional 2D transistor are still suffering low photosensitivity and volatile retention behavior, which can affect the recognition accuracy and long-term memory. Here, a novel optoelectronic synaptic device based on surface-state-rich CdSe nanobelt photosensitized 2D MoS_2_ transistor is demonstrated. Benefiting from the excellent light absorption of CdSe and effective charge trapping at the hetero-interface, the device exhibits not only high photosensitivity but also long retention time (>1,500 s). In addition, typical synaptic functions including the excitatory postsynaptic current, paired-pulse facilitation, the transformation from short-term to long-term plasticity, the transformation from short-term to long-term plasticity, spike-amplitude-dependent plasticity, and learning-forgetting-relearning process are successfully simulated and modulated by light stimulation. Most importantly, an artificial neural network is simulated based on the optical potentiation and electrical habituation characteristics of the synaptic devices, with recognition accuracy rates of 89.2, 93.8, and 91.9 % for file type datasets, small digits, and large digits are achieved. This study demonstrates a simple and efficient way to fabricate highly photosensitive optoelectronic synapse for artificial neural networks by combining the merits of specific materials and device architecture.

## 1 Introduction

Inspired by human brain, artificial neuromorphic computing has emerged as a promising approach to overcome the von Neumann bottleneck. As the basic unit of neuromorphic computing system, synapses play significant role in transmitting information between two neurons, designing artificial synaptic devices is the key step to construct brain-like computers [1]. Compared to the traditional electric-stimulated synaptic devices, the light-stimulated synaptic devices (optoelectronic synapse) that driven by optical signals to realize synaptic functions offer the advantages of high bandwidth, low cross-talk, and low power consumption [1], [2]. More importantly, the optoelectronic synapses have great opportunity in artificial visual perception system as they could detect light signals, store and process data in the same device [2], [3], [4].

Up to now, various optoelectronic synaptic devices based on perovskites [5], [6], organic materials [7], [8], nanowires [2], amorphous oxide semiconductors [9], [10], and transition metal dichalcogenides (TMDCs) [11], [12], [13], [14] have been extensively investigated to emulate the function of biological synapses. Among them, TMDCs are especially worth exploiting because of their excellent electric field tunability, remarkable optoelectronic conversion efficiency, and easy integration with other dimensional materials [13], [14]. As reported, some devices based on 2D/2D vdWHs have been shown enhanced synaptic performance. Wang et al. demonstrated the artificial synapse transistor based on a fully 2D inorganic/organic (MoS_2_/PTCDA) hybrid heterostructure, and the carriers transfer at the heterojunction interface results in an excellent synaptic plasticity [15]. Yang et al. reported the Bi_2_O_2_Se/graphene hybrid structures for applications as photodetectors, neuromorphic devices, and digital logic operations. By using the positive and negative photoresponse characteristics induced by the excitation wavelength, major synaptic functions are achieved [16]. Due to the narrow bandgap and strong absorption of near-infrared (NIR) light of Bi_2_Se_3_, Wang et al. fabricated the MoSe_2_/Bi_2_Se_3_ heterojunction for mimicking hetero-synaptic plasticity, and NIR was used to control the device’s conductivity and synaptic plasticity [17]. Chen et al. combined 2D ferroelectric α-In_2_Se_3_ with p-type GaSe as the active layer to form a synapse device. Pavlovian conditioning was mimicked by combining light pulses with electrical pulses [18]. Although great progress has been achieved, the poor light absorption of atomic thick TMDCs that results in limited light–matter interactions [19], [20] and inferior photoresponse performance based on conventional 2D/2D vdWHs, thus requiring high-power light stimulus for programming operations. Furthermore, the long-term memory ability still faces many challenges with respect to neuromorphic applications [21], [22]. This is largely due to the rapid detrapping behavior of the trapped photocarriers at the 2D/2D heterojunction interface. To address the above challenges, the integration of surface-state-rich photosensitive materials with TMDCs has been identified as an effective approach to construct high-performance phototransistor synapse [23], [24], [25], [26]. As they could improve the light absorption, enhance photocarrier separation efficiency, and enlarge the application range. Furthermore, the defects on the surface of non-layered 2D materials generate localized electronic states, which could serve as charge-trapping centers to trap electrons or holes and thus improve the synaptic properties. Therefore, it needs to investigate the specific optical materials and optimized device structure to simulate synaptic plasticity behavior under optical stimulation.

In this work, we designed and fabricated an optoelectronic synaptic device based on CdSe nanobelt/MoS_2_ vertical vdWH transistor, where CdSe and MoS_2_ respectively act as the photosensitive and channel layer. The interaction between CdSe and MoS_2_ enhances the separation efficiency of the photoexcited carriers, thereby improving the photoresponsivity and synaptic plasticity of the device. In addition, the introduction of CdSe induces delayed decay of the photocurrent in MoS_2_, resulting a long retention time exceeding 1,500 s under 405 nm illumination, which serves as the basis for emulating essential synaptic behaviors, including excitatory postsynaptic current (EPSC), paired-pulse facilitation (PPF), short/long-term memory (STM/LTM), and learning-forgetting-relearning process under light stimulation. Most importantly, based on the optical potentiation and electrical habituation properties of the device, an artificial neural network (ANN) is stimulated based on the CdSe/MoS_2_ synapse with maximum image recognition accuracy up to 91.9 %.

## 2 Experimental section

### 2.1 Fabrication of MoS_2_ transistor

The MoS_2_ transistor was prepared by a dry transfer method [27]. A large-area multilayered MoS_2_ nanosheet was mechanically exfoliated on top of the PDMS film and transferred onto the SiO_2_ (300 nm)/Si substrate on a transfer platform equipped with an optical microscope (Shanghai OnWay Technology Co., Ltd). Next, the source and drain electrodes were patterned by a maskless ultraviolet lithography machine (TuoTuo Technology (Suzhou) Co., Ltd.). Then Au (ZhongNuo Advanced Material (Beijing) Technology Co., Ltd) metal contacts with a thickness of 40 nm were deposited by using thermal evaporation with a lift-off process in acetone.

### 2.2 Preparation of CdSe nanobelts

CdSe powder placed in the central area of the tube furnace was used as the source material. Some chips of SiO_2_ (300 nm)/Si coated with 10 nm-thick Au were used as the growth substrates and placed in the downstream area of the tube furnace. Prior to the growth process, the quartz tube was pumped to vacuum by a pump. Afterwards, the high-quality Ar was introduced into the quartz tube and the flow rate was maintained at 100 sccm. Then, the temperature of the tube furnace was increased to 850 °C and kept for 40 min. After the reaction, the black products would be synthesized on the substrates.

### 2.3 Fabrication of CdSe nanobelt photosensitized MoS_2_ transistor

The cured polyvinyl alcohol (PVA) was attached to PDMS to form a PDMS/PVA stamp. The prepared CdSe nanobelts were transferred to a new SiO_2_/Si substrate with a contact printing method. The selected CdSe flake was picked up with the PDMS/PVA stamp and then aligned and precisely laminated on top of MoS_2_ channel without contacting the metal electrodes. As a result, a CdSe/MoS_2_ vertical heterojunction was formed.

### 2.4 Material and device characterization

Raman spectroscopy was measured by using a Horiba Raman system with 532 nm laser excitation to analyze the heterojunction. Local contact potential difference (CPD) between CdSe and MoS_2_ constructed on ITO substrates was investigated with KPFM. A commercial AFM (Dimension Icon, Bruker) in peak force mode KPFM was used to carry out morphology and surface potential measurements. The topographic imaging was performed in tapping mode. After that, the surface potential images were acquired with the tip lifted to a height of about 20 nm. In the KPFM measurements, a bias was applied on the tip, with a scan rate of 0.5 Hz. Tips of KPFM measurements coated Pt/Ir layer with a typical tip radius of ∼20 nm. All the electrical properties and biological synaptic characteristics of the device were measured at room temperature using a probe station equipped with a microscope and a B1500A semiconductor parameter analyzer (Keysight). Optical signals were provided by laser source with 405 nm wavelength and the radius of the laser spot is about 0.2 cm.

## 3 Results and discussion

**Figure 1:**
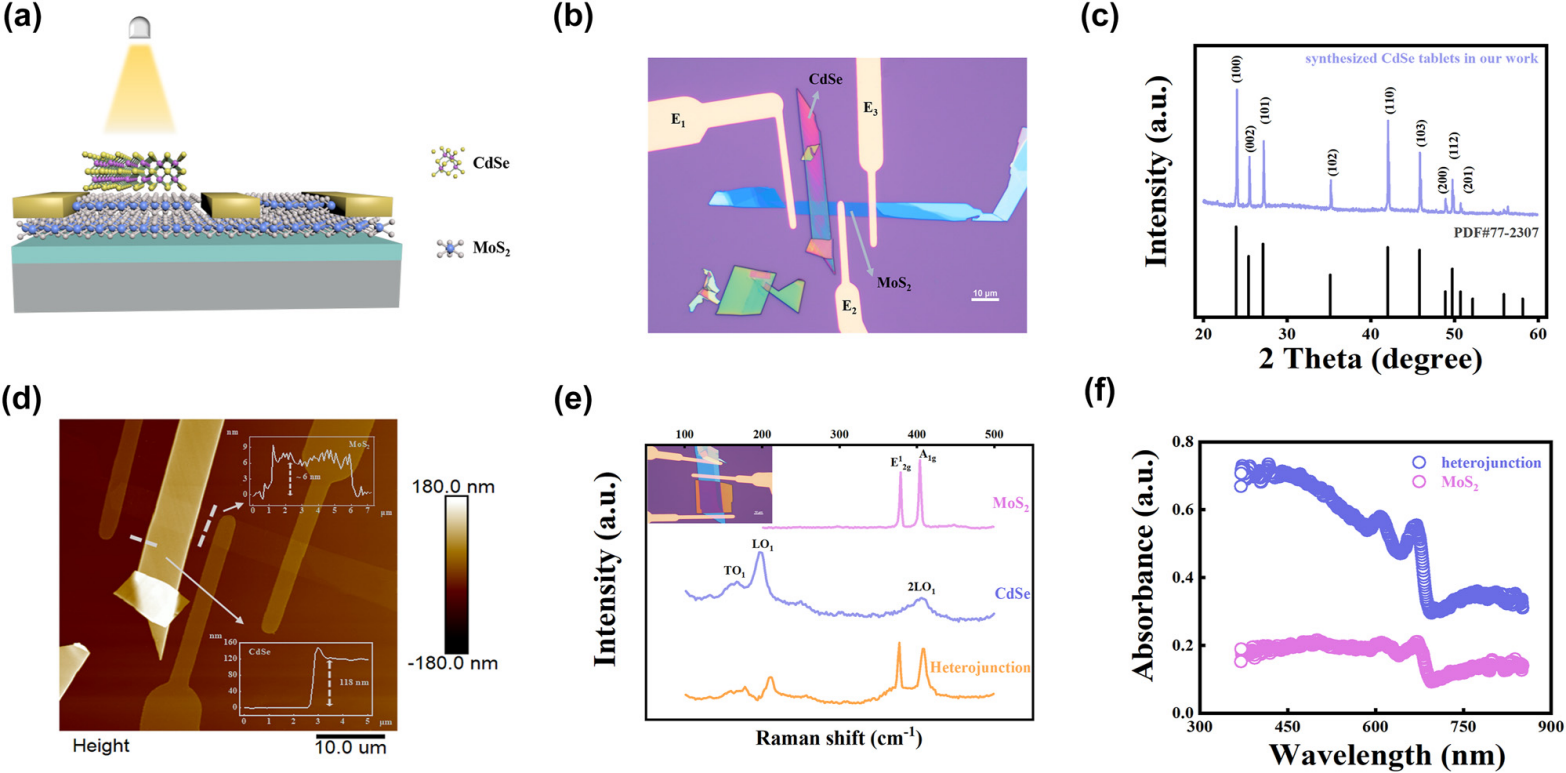
Design and characterization of CdSe/MoS_2_ device. (a) Schematic diagram of the CdSe/MoS_2_ vdWH device. (b) Optical microscopic image of the device. (c) XRD pattern of CdSe synthesized by PVD and comparison with standard card. (d) AFM image of CdSe/MoS_2_ device. (e) Raman spectrum of CdSe and MoS_2_ under 532 nm laser excitation. (f) UV–vis absorption spectra of MoS_2_ nanosheets, CdSe nanobelt, and CdSe/MoS_2_ heterojunction.

The CdSe nanobelt photosensitized MoS_2_ transistor was fabricated on a SiO_2_/Si substrate via PVA-assisted transfer method. Figure 1a and b show the schematic diagram and optical microscopic image of the CdSe/MoS_2_ heterojunction transistor, respectively. As shown in Figure S1, the physical vapor deposition (PVD) synthesized CdSe nanobelts have a width of a few micrometers, length of tens of micrometers. The XRD pattern is shown in Figure 1c, and all diffraction peaks can be indexed to the hexagonal structure of CdSe [28]. The thickness of MoS_2_ and CdSe measured by AFM is about 6 and 118 nm, respectively (Figure 1d). In order to characterize the optical properties of each layer and interface quality, Raman and UV–vis absorption were performed. The Raman spectrum of individual MoS_2_ and CdSe are shown in Figure 1e. Two obvious Raman peaks are observed at 378 cm^−1^ and 403 cm^−1^ for MoS_2_. In the Raman spectrum of CdSe, three distinct peaks presented at about 167 cm^−1^, 196 cm^−1^ and 406 cm^−1^, respectively. These Raman peaks are in consistent with previously reported data [29], [30]. For the CdSe/MoS_2_ heterojunction, all the above Raman peaks maintained and no noticeable peak position shift was observed, confirming the formation of high interface quality after the device fabrication. In Figure 1f, MoS_2_ has a low absorption in the visible range, and CdSe nanobelt exhibits strong light absorption in the range of 380–750 nm, covering almost the whole visible light region, while the absorption intensity of CdSe/MoS_2_ heterojunction is greatly enhanced compared to individual MoS_2_ and CdSe. This suggests that CdSe can act as an absorption layer of visible light to enhance the photoresponsivity and synaptic plasticity.

**Figure 2:**
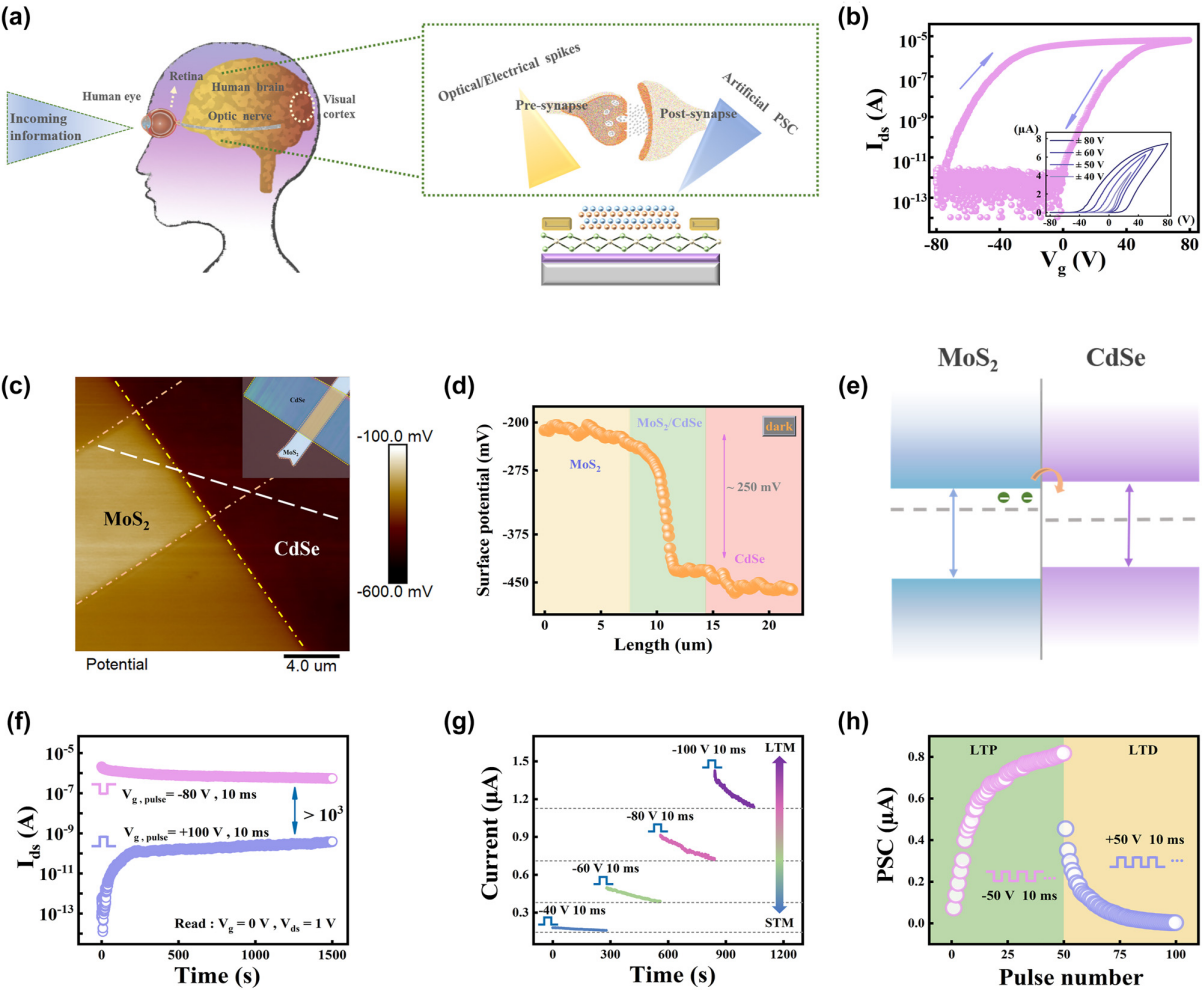
The electrical characterization of the CdSe/MoS_2_ device. (a) The left schematic is the human visual system. The right shows the comparison between biological synapse and the CdSe/MoS_2_ optoelectronic synapse. (b) Transfer curve of the CdSe/MoS_2_ device in dark at *V*_d_ = 1 V. The inset is the double-sweeping transfer curves with different sweeping ranges. (c) The KPFM image of the heterostructure. The inset is the optical image of the device based on ITO substrate. (d) The surface potential profile along the white line in the KPFM image. (e) The band alignment of the CdSe/MoS_2_ heterostructure. (f) The time-resolved retention performance of the CdSe/MoS_2_ after applying a positive and negative *V*_g_ pulse with 10 ms duration. (g) The EPSC excited by negative *V*_g_ pulses with different amplitudes, which shows the transition from STM to LTM behavior. (h) Long-term potentiation/depression characteristics for the CdSe/MoS_2_ transistor.

Figure 2a schematically depicts the diagram of the human visual nervous system. As external visual information coming from an object was focused onto the retina, the information is transmitted through the optic nerve and processed in the visual cortex [31]. The brain constantly receives, analyzes, stores information and makes decisions to achieve a variety of complex functions, depending on the synapse that exists between neurons. As shown in the right picture of Figure 2a, in biological neural network, the synapse is the connection between a pre-synaptic and a post-synaptic neuron. An external stimulus induces an action potential at pre-synaptic terminal, making the neurotransmitters released by pre-synaptic membrane and dock with the receptors on the post-synaptic neuron, resulting in the generation of excitatory post-synaptic current (EPSC) or inhibitory post-synaptic current (IPSC) in postsynaptic neuron. When the external stimulus is removed, the neurotransmitters in the post-synaptic membrane will gradually disappear, and thus the post-synaptic current slowly returns to its original state. This behavior is responsible for learning and memory behavior for synapse [7]. Inspired by the brain and visual nervous system, the CdSe/MoS_2_ phototransistor can be used to simulate the function of biological synapses. By analogy with biological synapses, the gate voltage or the laser stimulation can be regarded as a presynaptic input, while the source/drain electrodes serve as the postsynaptic output terminal, and channel conductance is defined as the synaptic weight.

Figure 2b shows the ambient double-sweep transfer curve of a typical CdSe/MoS_2_ transistor at *V*_ds_ = 1 V under dark condition. The device exhibits a typical n-type transport behavior with a high on/off ratio (≈10^7^). Moreover, as shown in the inset, the hysteresis window increases with the incremental of gate voltage sweeping, and a maximum window of 75 V was observed under −80 to 80 V sweeping. In comparison, the bare MoS_2_ transistor without CdSe layer showed a much smaller hysteresis window (Figure S2a), indicating a lower trapped charge density compared to the CdSe/MoS_2_ heterojunction. Previous studies have demonstrated the hysteresis can be attributed to the interfacial traps at the MoS_2_/SiO_2_ and the intrinsic defects in MoS_2_ [32], [33], [34]. Due to the same batch of SiO_2_/Si substrates and similar thickness of MoS_2_ were used, the larger hysteresis window of CdSe/MoS_2_ device can be attributed to interaction between CdSe and MoS_2_.

To investigate the band alignment between CdSe and MoS_2_, the Kelvin probe force microscopy (KPFM) mapping image of this vdWH is measured in dark and the result is shown in Figure 2c. The contact potential difference (CPD) between the sample and the KPFM tip is calculated by the following equation [35]:

$$CPD_{\text{Mo}{\text{S}}_{2}}=\frac{W_{\mathrm{Tip}}-W_{\text{Mo}{\text{S}}_{2}}}{-e}$$

$$CPD_{\text{CdSe}}=\frac{W_{\mathrm{Tip}}-W_{\text{CdSe}}}{-e}$$

where **W**_**tip**_, **W**_**MoS_2_**_, and **W**_**CdSe**_ are the work function of the tip (Pt/Ir-coated Si), MoS_2_, and CdSe, respectively. The variation in CPD between MoS_2_ and CdSe is given by

$$\Delta \text{CPD}=\text{CP}{\text{D}}_{\text{Mo}{\text{S}}_{2}}-\text{CP}{\text{D}}_{\text{CdSe}}=\frac{W_{\text{CdSe}}-W_{\text{Mo}{\text{S}}_{2}}}{-\text{e}}=250\;\text{mV}$$

Figure 2d presents the variation of the surface potential along the dashed line across the heterojunction region in Figure 2c. It can be seen clearly observed that the surface potential of MoS_2_ is ∼250 mV larger than that of CdSe. Therefore, the Fermi level of MoS_2_ is 250 meV higher than that of CdSe.

Based on the KPFM results and previously reported band properties of CdSe and MoS_2_ [36], [37], the energy band alignment is graphically described in Figure 2e. A type-II band alignment is formed between CdSe and MoS_2_, which is consistent with that calculated theoretically based on density functional theory (DFT). The calculation details and results are shown in Figures S3 and 4. Since the *E*_f_ of MoS_2_ is higher than that of CdSe, some electrons in MoS_2_ will diffuse to CdSe after contact and be trapped by the defects in CdSe. These electrons, together with that trapped at MoS_2_/SiO_2_ interface, serve as the local gate and result in a larger hysteresis window relative to pristine MoS_2_.

Figure 2f shows the retention properties of the CdSe/MoS_2_ device that record after an individual *V*_g_ pulse at *V*_ds_ = 1 V. When a positive voltage pulse (100 V, 10 ms) was applied to the gate electrode, the electric field induced a specific number of electrons in MoS_2_ were captured by the traps at the MoS_2_/SiO_2_ and the intrinsic defects in MoS_2_ (Figure S5a). At the end of the pulse, the channel current, namely, post-synaptic current (PSC), was driven to a low level of 0.06 pA due to the electrons trapped in MoS_2_ and at SiO_2_/MoS_2_ interface acted as local gate (Figure S5b). As exhibited in Figure 2f, the inhibitory post-synaptic current (IPSC) is followed by an increase process of ∼200 s before stabilizing at 118 pA, which may be due to some trapped electrons relaxed back to MoS_2_, so that the carrier density in the channel increases. On the other hand, when a negative pulse (−80 V, 10 ms) was applied to gate, the negative voltage pushed the electrons trapped at the MoS_2_/SiO_2_ interface and the defects of MoS_2_ into the MoS_2_ channel (Figure S5c). After the *V*_g_ was removed, the strength of local gate was reduced and MoS_2_ was switched to the high-conductance state (Figure S5d), and thus a high excitatory postsynaptic current (EPSC) of 2.12 µA was obtained, resulting in a stable on/off current ratio over 10^3^ was achieved for 1,500 s. The charge trapping and de-trapping process in CdSe/MoS_2_ device offers the basic condition for mimicking the synaptic functionality. The similar retention change has also been observed in bare MoS_2_ devices in Figure S2b. Due to the lack of the CdSe layer for trapping electrons, the local gate after positive *V*_g_ pulse is smaller than that in CdSe/MoS_2_ device, thus the IPSC is two orders of magnitude larger than that for CdSe/MoS_2_ device, and the on/off current ratio was reduced to 10 for pristine MoS_2_ transistor (Figure S2b).

In biological systems, short-term memory (STM) only lasts for seconds or tens of minutes, while long-term memory (LTM) can last from a few hours and even to a lifetime [26]. Figure S6 shows a schematic diagram of the memory consolidation process in the human brain. The STM can transform into LTM through increasing learning times or intensity. In this CdSe/MoS_2_ device, the transition from STM to LTM was successfully imitated by changing the amplitude of gate voltage. Figure 2g shows the time-dependent postsynaptic current (PSC) induced by a negative voltage pulse with same width of 10 ms but different amplitudes. The PSC increases immediately after a −40 V gate pulse and decays to the initial value after 300 s. This behavior is similar to the STM in our brain. As the amplitude of the gate pulse is increased, an LTM was achieved, which is characterized by the increased PSC and decay time than that of the −40 V ones. This is because stronger input pulse could drive more trapped electrons into the channel, leading to a higher EPSC, and the decay of EPSC would continue before some free electrons got trapped again and achieved a balance.

Long-term synaptic plasticity is composed of long-term potentiation (LTP) and long-term depression (LTD) of synaptic weights, which is a significant constituent part of the control memory in hippocampal neurons [4]. LTP/LTD can be mimicked by applying high frequency spikes to enhance/reduce the synaptic transmission. To verify the LTP and LTD, 10/20/30/50 consecutive negative gate pulses (−50 V, 10 ms duration) and then 10/20/30/50 consecutive positive pulses (50 V, 10 ms duration) were applied to the gate electrode as presynaptic input, whereas drain current was continuously recorded as PSC. As shown in Figure S7a, the PSC could be modulated with a total of 10, 20, 30, and 50 cycles of *V*_g_ pulses. The PSC increases more rapidly in the first 20 negative *V*_g_ pulses and then slightly saturates as the pulse number increases to 30 and 50 (Figure 2h). In contrast, the PSC rapidly decreases and then saturates as the number of the positive *V*_g_ pulses increase, suggesting the capability of CdSe/MoS_2_ device to mimic the LTP and LTD behavior of a synapse. Figure S7b represents the PSC response to six successively decreasing negative *V*_g_ pulses (from −10 to −100 V) with a pulse duration of 10 ms, the PSC increases gradually due to more trapped electrons are driven to MoS_2_ layer, which can be used to mimic the potentiation of the synaptic strength. Afterward, under six successively increasing positive *V*_g_ pulses (from 10 to 100 V, duration: 10 ms), more electrons are trapped at the interfacial SiO_2_ layer and defects of MoS_2_, thus the conductance of the device decrease gradually, corresponding to the habituation of the synaptic strength. The EPSC and IPSC can also be adjusted by the pulse amplitude. The PSC under a sequence of ten consecutive gate pulses with amplitude varying from −10 to −100 V was recorded in Figure S8a. The change of PSC after each pulse is extracted and plotted in the inset. It can be observed that the current increased by four orders of magnitude from 2.89 × 10^−10^ A at −10 V to 2.27 × 10^−6^ A at −100 V for a same pulse width of 10 ms. Similarly, the synaptic inhibitory behavior was observed by applying consecutive positive *V*_g_ pulse, as shown in Figure S8b. The PSC continuously decreases as the pulse amplitude increases from 10 V to 100 V. Therefore, the CdSe/MoS_2_ device could respond to stimulation with different-amplitude to mimic the synaptic behavior of spike-amplitude-dependent plasticity (SADP).

**Figure 3:**
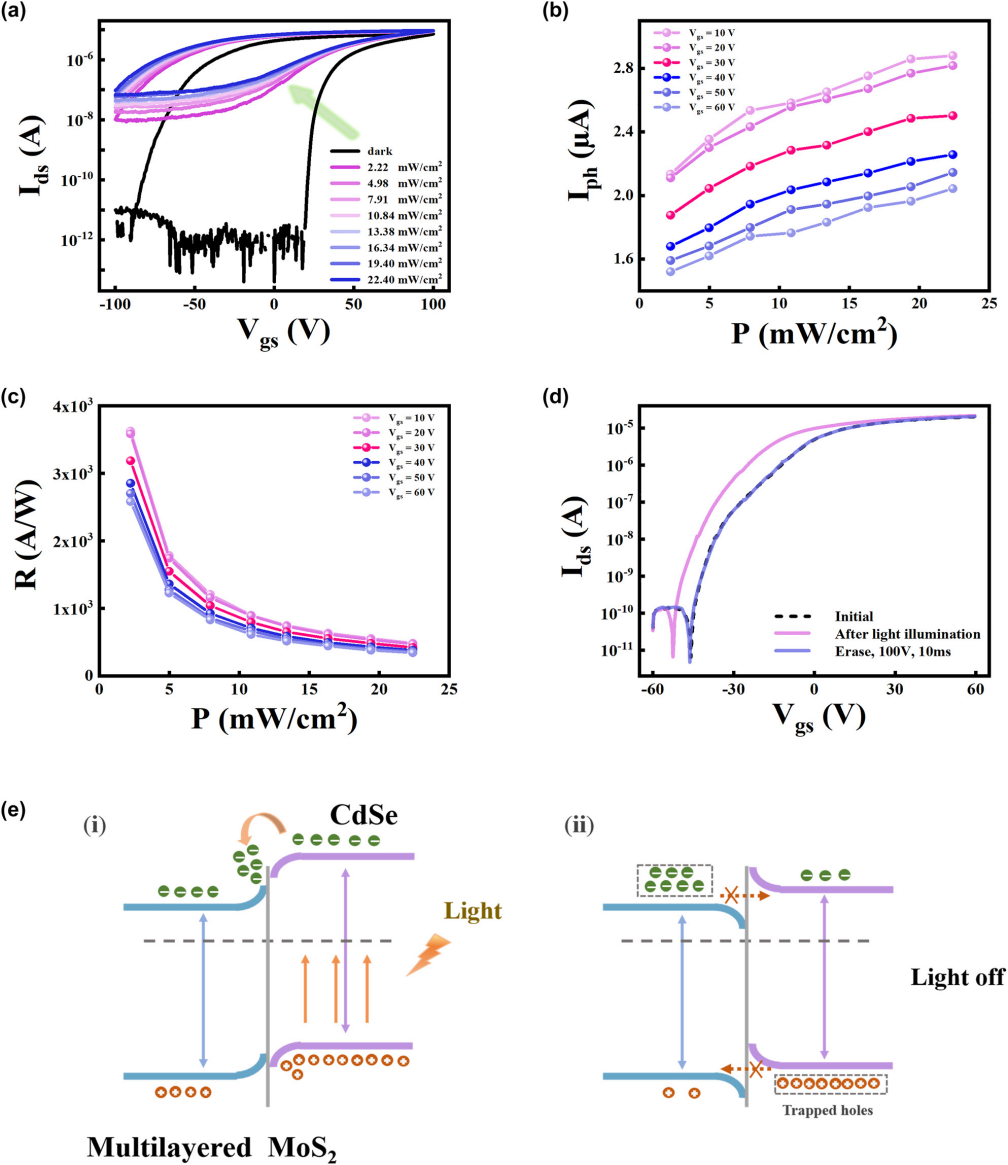
The energy-band diagrams of CdSe/MoS_2_ heterojunction and the transport of photogenerated carriers at the hetero-interface. (a) The double-sweep transfer curves of the device under dark condition and 405 nm laser with different power densities. (b) Photocurrent and (c) responsivity of the device under various light power intensities at different gate voltage. (d) The transfer curves of the CdSe/MoS_2_ device at initial state, optically programmed (450 nm, 10.84 mW/cm^2^ for 10 s) state and electrically erased (100 V for 10 ms) state. (e) Schematic diagram for charge generation and transport process at the CdSe/MoS_2_ interface under laser irradiation and after removing irradiation.

In addition to modulating excitatory and inhibitory synaptic behaviors with electrical pulses, the device can also effectively imitate the neuromorphic function with light stimulation, owing to the strong light absorption of CdSe. To investigate the photoresponse characteristics of the CdSe/MoS_2_ device, the transfer curves of the device under different light intensities (*λ* = 405 nm) are measured and shown in Figure 3a. Under light illumination, the *I*_ds_ exhibits an abrupt increment and left shift of the threshold relative to that in dark, which is caused by the increased charge carriers in MoS_2_ channel that mainly derived from the separation of photoexcited carriers at the CdSe/MoS_2_ hetero-interface. The photoresponsivity (*R*) of the device is plotted as the function of the power density according to the following equation [38]:

$$R=\frac{I_{\text{ph}}}{P_{\text{in}}\cdot A}=\frac{I_{\text{photo}}-I_{\text{dark}}}{P_{\text{in}}\cdot A}$$

where *I*_photo_, *I*_dark_, *P*_in_, *A* are photocurrent, dark current, light power intensity, and effective area of the device. As shown in Figure 3c, the *R* varies with the gate voltage, and a maximum value reaches 3.624 × 10^3^ A/W at 2.22 mW/cm^2^ and *V*_g_ = 10 V. This result indicates the CdSe/MoS_2_ heterojunction transistor is highly sensitive to visible light and has the potential to realize light stimulated synapse. Subsequently, the optically-programmable and electrically-erasable properties are investigated. Figure 3d shows the transfer curves of CdSe/MoS_2_ heterojunction before and after light illumination at *V*_ds_ = 1 V. An optical pulse (405 nm, 10.84 mW/cm^2^, width of 10 s) was used for programming operation, while electrical pulse with a height of −100 V and a width of 10 ms was employed as erasing operation. After the optical programming operation, the transfer curve shifts toward the left direction, while the threshold voltage (*V*_th_) can be restored to its initial state after the electrical erasing operation.

This phenomenon can be explained with band structure of the heterojunction. Due to the diffusion of electrons from MoS_2_ to CdSe after contact, a built-in electric field is formed directed from MoS_2_ to CdSe. As shown in Figure 3e, when light is illuminated on the device, a large number of electron-hole pairs are mainly photoexcited in the CdSe, and the electrons are quickly driven to the MoS_2_ by the built-in electric field, whereas the holes are confined in CdSe due to the energy barrier, resulting in a strong photogating effect even after removing the illumination, which leads to the negative shift of transfer curve. When electrical erasing operation (100 V, 10 ms) is applied, the electric field induces a specific number of electrons trapped at the MoS_2_/SiO_2_ interface, and thus the electron concentration in MoS_2_ channel is reduced and the Ids is restored to its initial state again.

**Figure 4:**
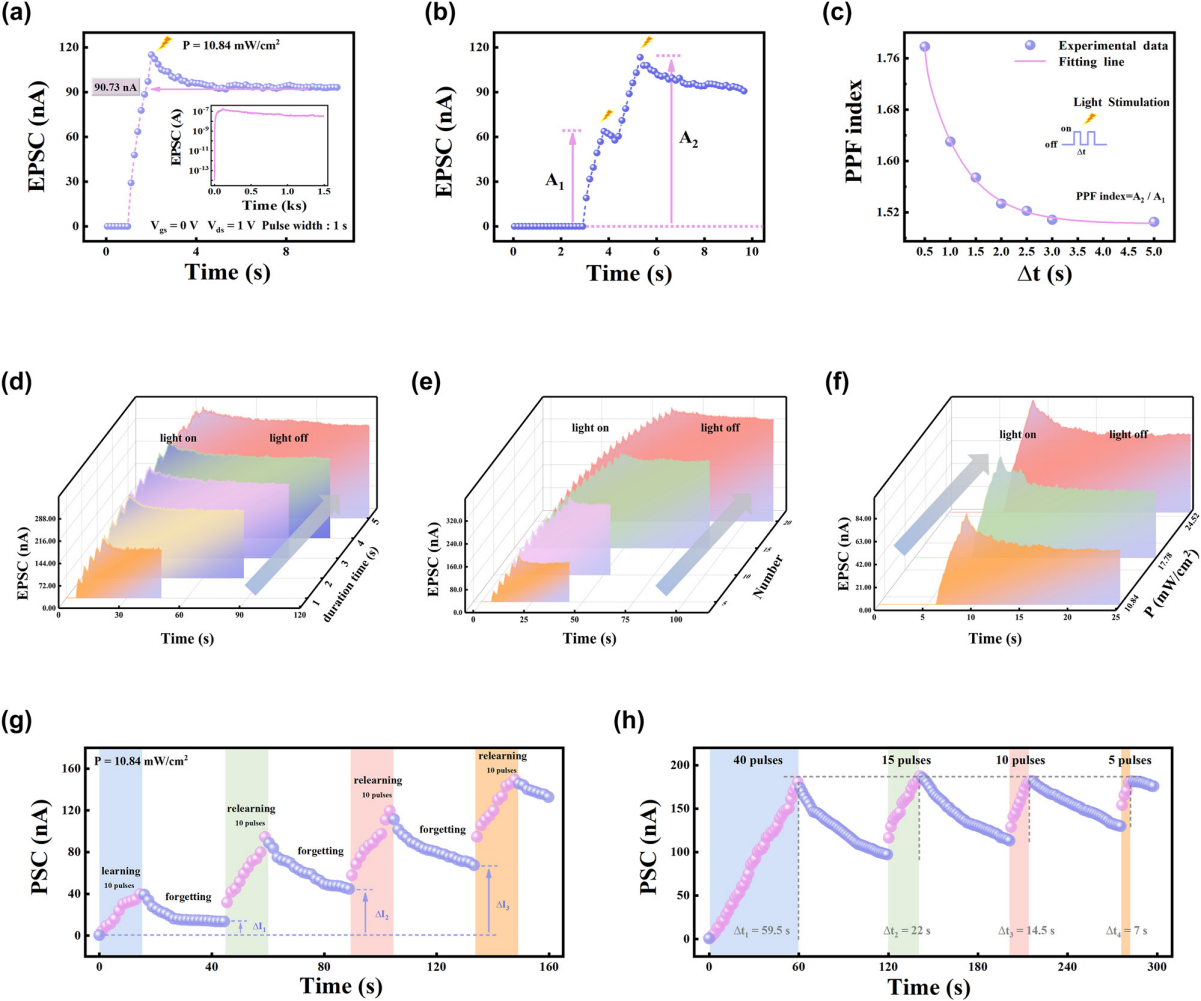
Optical synaptic behavior of the fabricated device. (a) The transient EPSC response triggered by a light spike (*λ* = 405 nm, 1 s, 10.84 mW/cm^2^). The inset is the long-memory retention performance of the transistors after removal of light source. (b) EPSC of the device excited by two adjacent laser pulses with an interval of 0.5 s. (c) The PPF index is plotted as a function of pulse interval time. The transition from short-term memory to long-term memory under successive light pulses by changing the (d) width, (e) number, and (f) intensity of light pulse using 405 nm laser (the reading voltage is fixed at 1 V). (g) The “learning-forgetting” process of four cycles. (h) The demonstration of the “learning-forgetting-relearning” process with the learning and relearning process reach up to the same level.

To investigate the learning and memory behavior of the CdSe/MoS_2_ synaptic device, Figure 4 shows the time-dependent photoresponse of the device at *V*_ds_ = 1 V after individual light pulse stimulation. As shown in Figure 4a, upon laser illumination (405 nm, 10.84 mW/cm^2^, duration = 1 s), the *I*_ds_ rapidly increases up to 115.1 nA, corresponding to the EPSC behavior of biological synapse. When light illumination is removed, the *I*_ds_ slowly decays to 90.73 nA during the first 2 s and then stabilizes at this value. The current level is much higher than the initial-state current of 1.14 × 10^−12^ A, indicating a memory effect towards the light stimulus. This charge storage behavior is analogous to the long-term plasticity of biological synapses, which is prerequisite to ensure reliable study on the synaptic functions. A long retention time is required for achieving long-term memory. The memory retention time of the CdSe/MoS_2_ device was measured with the device trained by ten light pulses, where the EPSC decayed from 172 to 40 nA within 1,500 s after removing the light pulses. While the current for pristine MoS_2_ transistor is reduced from 22 to 2.6 nA within 32 s (Figure S9). The longer retention time for the CdSe/MoS_2_ device can be ascribed to the introduction of CdSe layer. When a laser pulse is applied, a large number of electron-hole pairs were generated in CdSe due to its excellent optical absorption properties, and then the electrons were transferred to MoS_2_ due to the built-in potential, resulting in a surge of PSC and negative shift of *V*_th_. While the holes are confined to CdSe due to the energy barrier, leading to a strong photogating effect, which increases the electron concentration in MoS_2_ channel. After removing the light stimulus, the slow recombination of electrons and holes induces the channel exhibiting a long-term conductive, which accounts for the slow conductance decay behavior for the CdSe/MoS_2_ synaptic device. This is a prerequisite for reliable study of synaptic photoresponse.

PPF is a typical short-term synaptic plasticity triggered by two consecutive presynaptic spikes, which is essential to decode temporal vision or auditory information in biology neural systems. It means that when two consecutive stimuli with a short time interval are applied, the amplitude of current changes caused by the second stimulus is larger than that caused by the first one [10]. The PPF behavior can be mimicked by applying two consecutive light stimulation with an interval time (Δ*t*) to the CdSe/MoS_2_ device. As shown in Figure 4b, the amplitude of PSC triggered by the second laser pulse is significantly higher than that stimulated by the first, which is very similar to the PPF behavior in the biological synapses. The PPF index can be described as [19]:

$$PPF\;index=\frac{A_{1}}{A_{2}}\times 100\%$$

where *A*_1_ and *A*_2_ represent the amplitudes of the PSC triggered by the first and second light pulses. The dependence of the PPF index on Δ*t* is shown in Figure 4c. With the increases of Δ*t*, the PPF index gradually decreases, and reaches a maximum value (178 %) at 0.5 s, suggesting the memory formation process can be accelerated by reducing the interval between stimulus. It is noteworthy that the PPF value is comparable to that of most previously reported optoelectronic synapse devices [19], [39] (Table 1). This is because the trapped photogenerated holes inside the CdSe layer need to take a long time to decay. Pink curve shows the fitting result of the decay trend by a double exponential function [39]:

$$PPF\;index=C_{0}+C_{1}\times \exp\left(-\frac{\Delta t}{\tau_{1}}\right)+C_{2}\times \exp\left(-\frac{\Delta t}{\tau_{2}}\right)$$

where *τ*_1_ and *τ*_2_ represent the characteristic relaxation times of the rapid and slow phases. The fitting results show that *τ*_1_ and *τ*_2_ are 37 and 773 ms, respectively, and both values are close to that measured in the biological synapses (*τ*_1_ = 40 ms, *τ*_2_ = 300 ms) [40]. This feature indicates that learning and memory are associated with frequent and repetitive training.

Here, the transition from STP to LTP are further emulated in the CdSe/MoS_2_ device by altering light spike duration, number, and intensity, and these parameters can be deemed as the learning time, number, and intensity. Figure 4d shows the time-dependent EPSC characteristics of CdSe/MoS_2_ device under five laser spikes with different width. Clearly, as the spike width increases from 1 to 5 s, the cumulative spikes induced EPSC is increased by 4 times, and the decay time is prolonged and the stable current is increased, which indicates the transition of the synaptic plasticity from STM to LTM under the cumulative stimulation. Besides the spike width, as presented in Figure 4e and f, the number and intensity of light spikes can also be used to modulate the transition from STM to LTM. With the light pulse number (1–5) and pulse intensity (10.84–24.52 mW/cm^2^), the EPSC value gradually increased after the removal of light illumination, and the decay time of EPSC was prolonged. This phenomenon can be ascribed to the fact that longer illumination time (or stronger optical signals) result in higher carrier concentration in the MoS_2_ channel and more negative shift of *V*_th_ due to the enhanced photogating effect. This phenomenon is consistent with the Atkinson–Shiffrin’s memory model that the short-term memories can be transferred into long-term memory through repeated learning [41]. In addition, the light wavelength-dependent EPSC of the device are investigated and the results are shown in Figure S10. It can be observed that light illumination with short wavelengths generates the larger PSC of 145.97 nA for 532 nm and 160.98 nA for 670 nm at the end of the spikes, while near-infrared light irradiation (808 nm) produces a low current of 5.74 nA, which is consistent with the UV–vis absorption spectrum of CdSe. Such optoelectronic synaptic device architecture should be applicable to other vdWHs. Figure S11 shows the schematic diagram of the fabricated light-stimulated CdSe/ReS_2_ synaptic device. Under dark condition, the device also exhibits a large hysteresis memory window. When light pulses are applied to the device, some typical synaptic behaviors, such as EPSC, PPF, and STM-LTM, are also successfully emulated in this device. The detailed information can be found in Figure S11.

Based on the above synaptic behavior of the CdSe/MoS_2_ device, the “learning-forgetting-relearning-forgetting” experience of human brain is emulated in this device. Figure 4g presents four cycles of “learning-forgetting” processes. The first learning process was performed by applying 10 light pulses (405 nm, 10.84 mW/cm^2^, duration of 1 s) at Vds = 1 V, which results in the channel current significantly increasing from 2.27 pA to 39.39 nA. After removing the light illumination, the PSC gradually decays to 13.04 nA within 30 s. This process corresponds to the forgetting process. The CdSe/MoS_2_ device can retain 33 % of the memory in the end, which indicates that some part of the information learned by the human brain will be forgotten after a period of time. However, memory loss with time can be improved by repeated learning. The post-synaptic conductance at the end of the forgetting process, corresponding to the cognitive level, increased gradually with the number of learning-forgetting cycles as indicated by Δ*I*_3_ > Δ*I*_2_> Δ*I*_1_.

When 40 consecutive light pulses (*λ* = 405 nm, 1 s) are applied to the CdSe/MoS_2_ device during the first learning process, the PSC gradually increases from 0.16 to 180 nA (Figure 4h), indicating an increase in synaptic weights. Subsequently, the current decays to 96.8 nA after 60 s of forgetting process. In the second learning process, only 15 light pulses are needed to recover to the current level that the first learning process achieved, and this value further reduced to 10 and 5 in the third and fourth learning process, indicating that the relearning process becomes much easier. It can also be observed that the decay trend of the PSC slows down after relearning process. This trend is consistent with human’s “learning–forgetting–relearning–forgetting” model. It requires less time to relearn previously forgotten information than to learn for the first time, and timely review could reduce the forgetting rate and strengthen the memory capability. This result demonstrates the feasibility of the CdSe/MoS_2_ device in constructing the biological nervous system.

**Figure 5:**
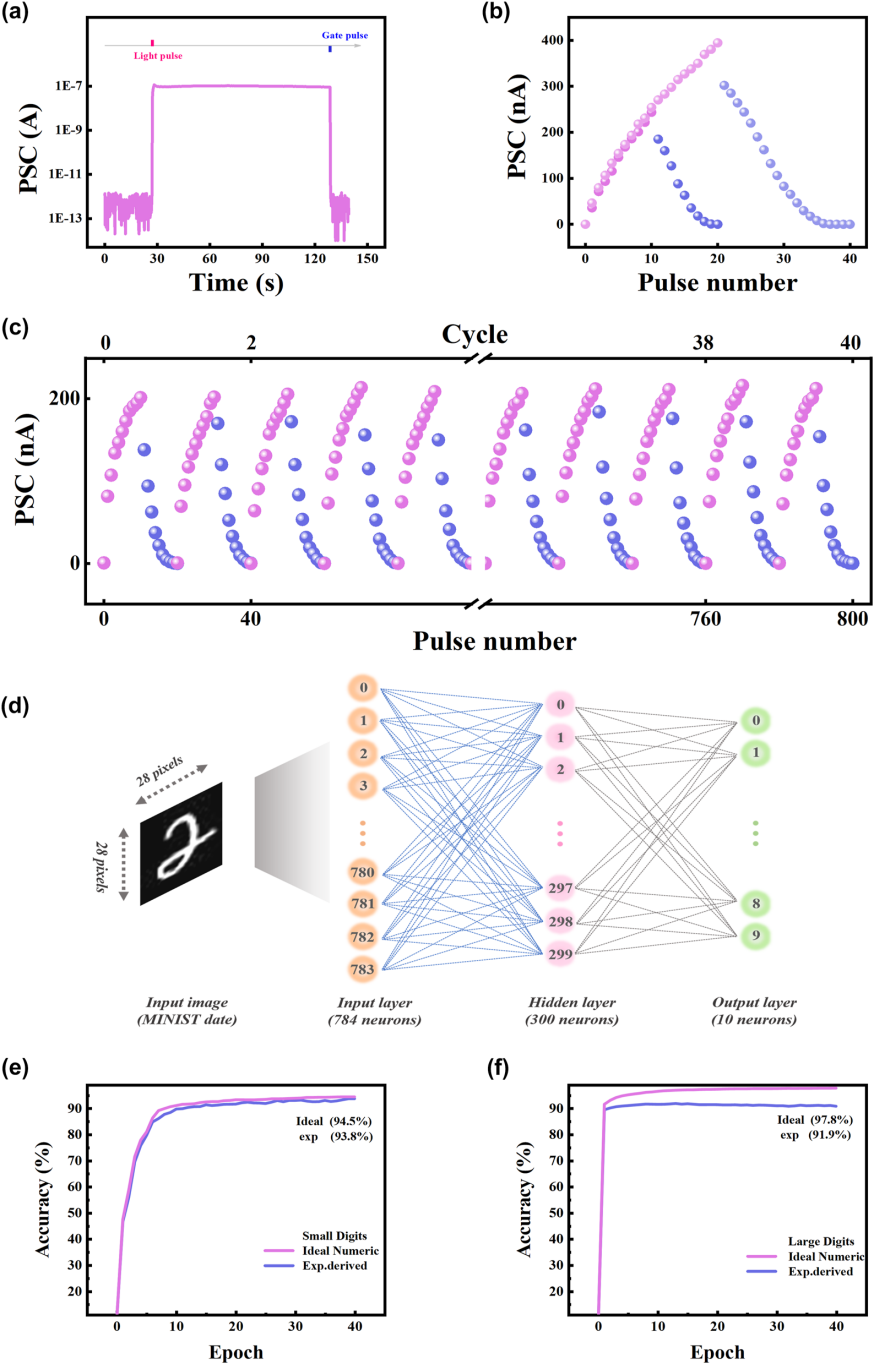
Simulation of artificial neural network for image recognition based on the CdSe/MoS_2_ synaptic device. (a) The programming and erasing operations of PSC by light pulse and gate voltage pulse. (b) Long-term potentiation/depression characteristics for the CdSe/MoS_2_ transistor triggered by 10/20 successive light pulses and then 10/20 gate voltage pulses. (c) The 40 cycles of long-term potentiation/depression curve based on CdSe/MoS_2_ synaptic transistor. (d) Schematic illustration of neural network for recognition of large image tasks. The simulated recognition rate as a function of training epochs for the (e) small image and (f) large handwritten digits.

Except for natural forgetting, applying a positive gate voltage spike (100 V, 1 s) could directly erase the PSC to the initial state of CdSe/MoS_2_ device, as precisely shown in Figure 5a. This is because the positive gate voltage can induce many electrons in MoS_2_ channel trapped at the MoS_2_/SiO_2_ interface, which may depress the device to the initial low conductance state. Despite the stable long-term potentiation/depression (LTP/LTD) have achieved with electrical programming/erasing (Figure 2h), the asymmetry between LTP and LTD may reduce accuracy of pattern recognition, which is not expected in neuromorphic computing [42]. To overcome the shortcoming, the optical stimulation induced LTP and electrical spikes driven LTD synaptic behavior were successfully emulated with CdSe/MoS_2_ transistor. As shown in Figure 5b, the current increases gradually with 10/20 consecutive optical pulses (405 nm, 10.84 mW/cm^2^, duration of 1 s), which represent the potentiation of the synaptic weight. Subsequently, the current decrease gradually with same number of electrical pulses (*V*_g_ amplitude uniformly increasing from 10 to 100 V, step = 10 V, pulse width of 10 ms), corresponding to the depression characteristics. The near-linear and symmetric potentiation-depression response is the basis of realizing high image recognition accuracy. To evaluate the endurance performance of potentiation-depression for the CdSe/MoS_2_ device, 40 cycles of photonic potentiation (1 s duration with 1 s interval) and electrical depression were tested, and the results shown in Figures 5c and S12a exhibit nearly less conductance variation between the first and last cycle, reflecting repeatable switching and good endurance of the device. During the entire 40 cycles measurement, the device is periodically switched between program and erase state, and the program/erase ratio is maintained above 10^4^ (Figure S12b).

The cycle-to-cycle reproducibility demonstrates high stability and switching endurance of the CdSe/MoS_2_ device. To evaluate the learning ability of CdSe/MoS_2_ synaptic devices, an artificial neural network (ANN) with a three-layer structure was constructed for handwritten digit recognition. As shown in Figure 5d, the network is consisting of the input layer (784 neurons), hidden layer (300 neurons), and output layer (10 neurons), where the input layer corresponds to 28 × 28 pixels input image from the “Modified National Institute of Standards and Technology” (MNIST) dataset, and the output layer corresponds to 10 categories that recognize the numbers 0 to 9. The ANN was trained with the back-propagation algorithm using three datasets: small images with 8 × 8 pixels of handwritten digits, large images with 28 × 28 pixels of handwritten digits from the MNIST dataset, and a Sandia file classification dataset. The conductance states of the CdSe/MoS_2_ synaptic transistor are used as a weight update for performing back-propagation based on the experimental results of the 40 cycles of optical potentiation and electrical depression curve. The pattern recognition accuracy of CdSe/MoS_2_ device and ideal synapse for file types, small and large digits are presented in Figures 5e, f and S12c, respectively. The simulation results of the artificial neural network under ideal conditions indicate the limit of algorithm accuracy, which is plotted by the pink curve. After 40 training epochs, the file types recognition accuracy based on CdSe/MoS_2_ device is 89.2 %, which is close to the ideal value (93.3 %). The recognition accuracy of small images reaches up to 93.8 %, which is almost the same as the ideal value (94.5 %). For the simulations of large images with 28 × 28 pixels also obtain a high accuracy of 91.9 %, which is comparable or higher than that of most reported optoelectronic synaptic devices [43], [44], [45] (Table 1). Such remarkable recognition accuracy can be attributed to the linear tendency of channel conductance. These excellent performances demonstrate the possibility of the CdSe/MoS_2_synaptic device for the construction of artificial neural networks in applications of neuromorphic computing.

**Table 1:** Comparison of recognition accuracy and PPF index among different artificial synaptic devices.

Materials	Wavelength (nm)	Pulse (s)	PPF (%)	Accuracy (%)	Epoch	Ref.
MoS_2_/PTCDA	532	0.4	147	NA	NA	[39]
Bi_2_O_2_Se/graphene	635	0.1	120	NA	NA	[16]
Cs_2_AgBiBr_6_/IGZO	365	30	206.9	83.8	18,000	[43]
Cs_3_Bi_2_I_9_/PMMA/DPPDTT	405	1	122	92.1	10,000	[46]
ZnO/PbS	365	0.2	46	67	4,000	[47]
BP flakes	280	10	NA	90	1,000	[44]
IGZO-alkylated/graphene	405	0.05	182	62	36,000	[48]
UCNPs-MoS_2_	980	5	190	70	15,000	[49]
WSe_2_/h-BN flakes	655	0.01	NA	90	50	[45]
CuPc/p-6P thin films	365	0.5	NA	78	5,000	[50]
CdSe/MoS_2_	405	1	178	91.9	40	This work

## 4 Conclusions

In summary, a highly photosensitive optoelectronic synaptic device is demonstrated based on CdSe nanobelt sensitized 2D MoS_2_ transistor. The device combines efficient light absorption layer, high mobility channel material, and efficient charge transfer, leading to excellent electric tunability and superior photoresponse characteristics. The photogating effect at the CdSe/MoS_2_ interface results in a long retention time exceeding 1,500 s, which provides the basis for achieving efficient light-stimulated synaptic behaviors, such as EPSC, STM-LTM, PPF, SADP, and learning–forgetting–relearning process. The synaptic potentiation and depression can be modulated under the stimulation of optical and specific electrical pulses, respectively. In addition, the recognition accuracy of handwritten digits with 28 × 28 pixels in MNIST dataset reaches up to 91.9 % after 40 training epochs by using a three-layer ANN, which is higher than most of reported optoelectronic synaptic devices. This work provides a simple and attractive strategy for the fabrication of optoelectronic synaptic devices for neuromorphic computational networks.

## Supporting Information

The characterization of the pristine MoS_2_ nanobelt and FETs, the details of the DFT calculation and the calculated band structure of CdSe/MoS_2_ heterojunction, schematic of interface traps that capture and release electrons, schematic diagram of the memory consolidation process in the human brain, performance of potentiation-depression with consecutive 4 sets of different electrical pulse numbers, the retention performance of bare MoS_2_ transistors, and the characteristics of the artificial optoelectronic synaptic device based on the CdSe/ReS_2_ heterojunction.

## Supplementary Material

Supplementary Material Details
