# Supplementary material for: Artificial optoelectronic synapse based on CdSe nanobelt photosensitized MoS2 transistor with long retention time for neuromorphic application
Source: Nanophotonics. 2024 Aug 29;13(22):4211–24. doi: 10.1515/nanoph-2024-0368 (PMC11501069; doi:10.1515/nanoph-2024-0368)
Supplement: Supplementary file 1 — Supplementary Material Details [file j_nanoph-2024-0368_suppl_001.docx]

**Supporting Information**

**Artificial Optoelectronic Synapse Based on CdSe Nanobelt Photosensitized MoS_2_ Transistor with** **Long Retention Time for Neuromorphic Application**

Xiaohui Song,^1^ Xiaojing Lv,^1^ Mengjie He,^1^ Xuan Qin,^1^ Fei Mao,^1^ Jie Bai,^1^ Yanjie Hu,^1^ Zinan Ma,^1^ Zhen Liu,^1^ Xueping Li,^2^ Chenhai Shen,^1^ Yurong Jiang,^1^ Xu Zhao,^1^ Congxin Xia^1,^[[1]](#footnote-0)^*^

*^1^Henan Key Laboratory of Photovoltaic Materials, Department of Physics, Henan Normal University, Xinxiang 453007, China*

*^2^Department of Electronic and Electrical Engineering,* *Henan Normal University, Xinxiang 453007, China*


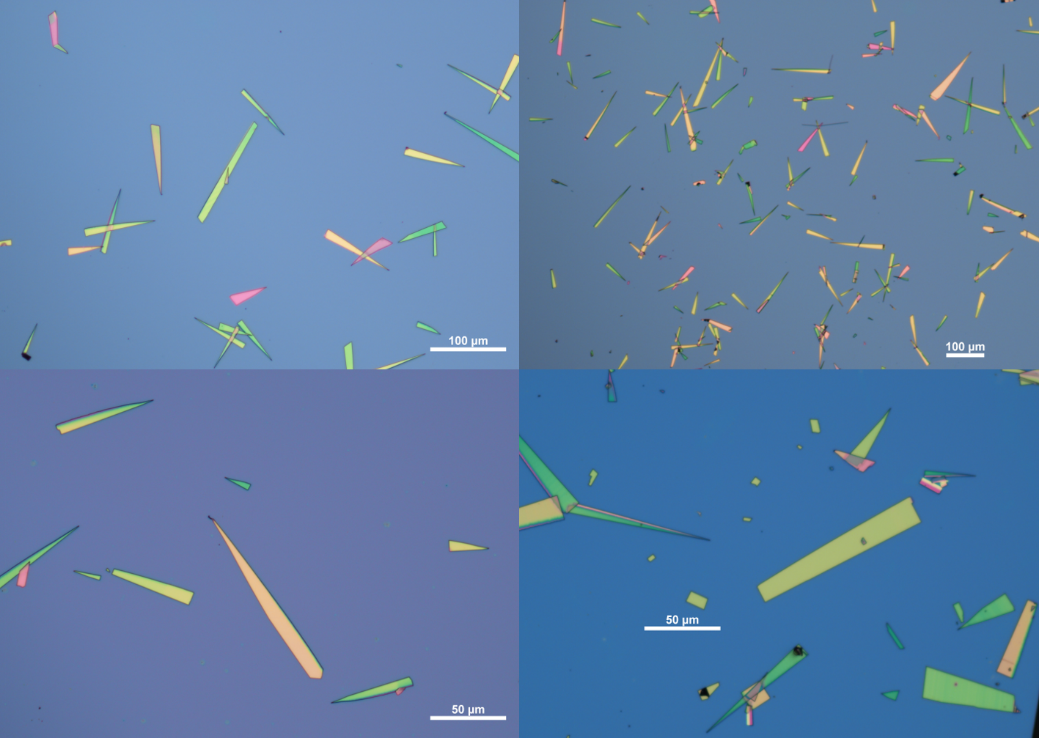


**Figure S1.** Optical microscopic image of the PVD synthesized CdSe nanobelts after contact printing.


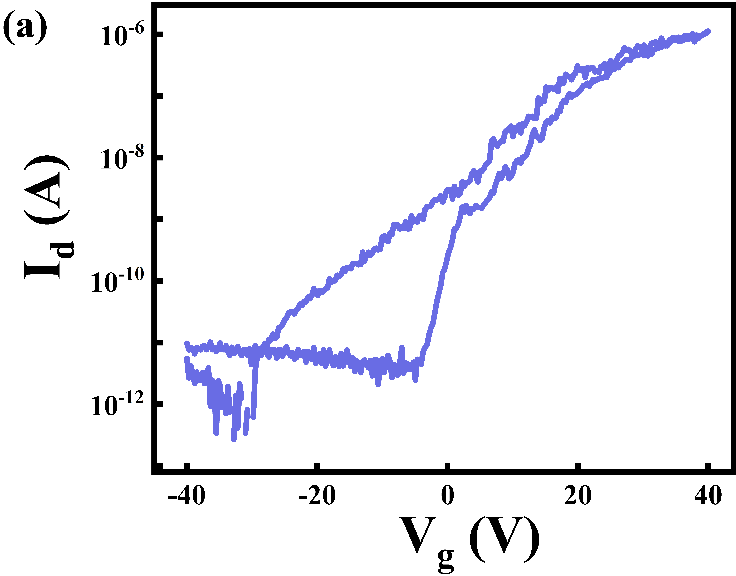

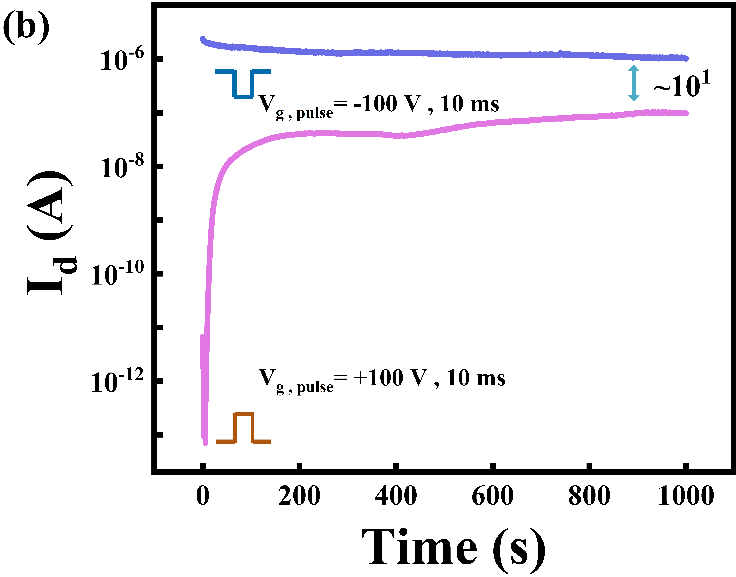


**Figure S2. The electrical characterizations of the pristine MoS_2_ FET.** **(a)** Typical dual-sweeping transfer curve of device. **(b)** The time-resolved retention performance of the drain-source current after applying gate voltage (Vg) pulse of -100 V and 100V with 10 ms duration in the dark, with V_writing_ of -100 V and 100 V separately.

**Note 1.** The density functional theory (DFT) calculation is carried out by the Vienna ab initio simulation package (VASP) with the projector-augmented wave (PAW) method to optimize the models and calculate their electronic properties. The generalized gradient approximation of Perdew–Burke–Ernzerhof (PBE) parametrization is used to describe the exchange-correlation potentials. The energy cutoff is set for 500eV. The force and energy convergence standards are 0.01 eV Å−1and 10^−5^ eV, respectively. The 13×13×1 k-points are sampled with the Monkhorst-Pack method for optimization and electronic structures. The DFT-D2 method is used to describe long-range van der walls (vdW) interactions and the dipole correction is applied in all calculations. Besides, the vacuum space greater than 20 Å along the z direction is set up for all models.

Here, the CdSe (110) supercell and MoS_2_ supercell are combined to construct the CdSe/MoS_2_ heterostructure model (Figure S8) with a lattice mismatch of about 1.17%, and the optimized lattice parameters is a=11.524 Å, b=8.48 Å. The optimized lattice parameters a= b = c =6.108 Å for CdSe and a=b=3.19 Å for MoS_2_.


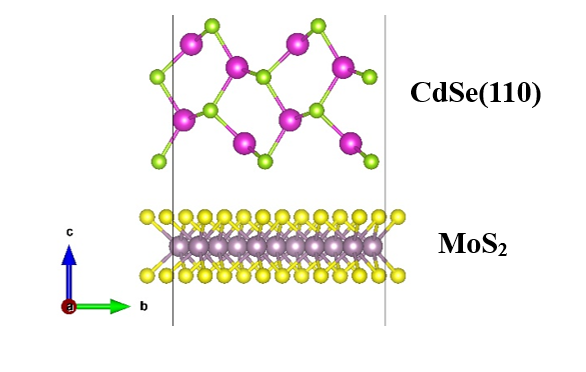


**Figure S3.** Optimized geometric structures of CdSe/MoS_2_ heterostructure.


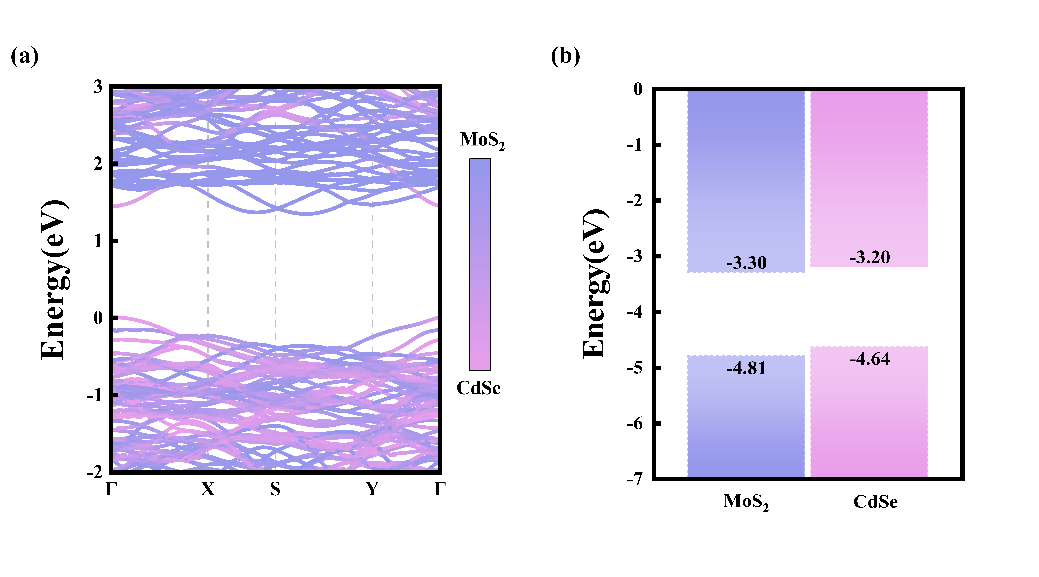


**Figure S4.** The calculated band structure (a) and the band alignment (b) of CdSe/MoS_2_ heterojunction.


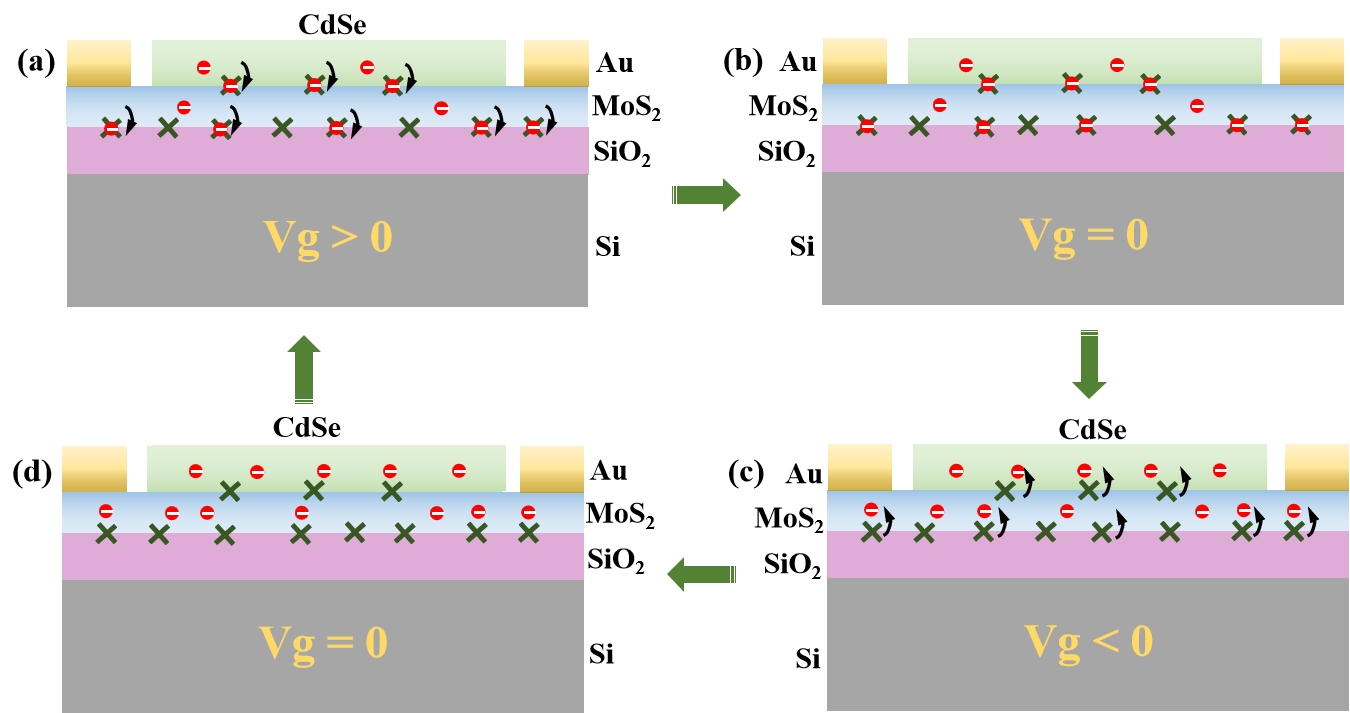


**Figure S5. Schematic of interface traps that capture and release electrons.** **(a)** When a positive gate pulse is applied, the electrons are captured by the interface traps between MoS_2_ and SiO_2_ substrate. **(b)** When the positive gate pulse is removed, the channel is driven to a low conductance state due to the trapped electrons acted as a local gate. **(c)** A negative gate pulse releases electrons trapped at the SiO_2_/MoS_2_ interface into the MoS_2_ channel. **(d)** At the end of negative gate pulse, a high conductance channel was achieved.


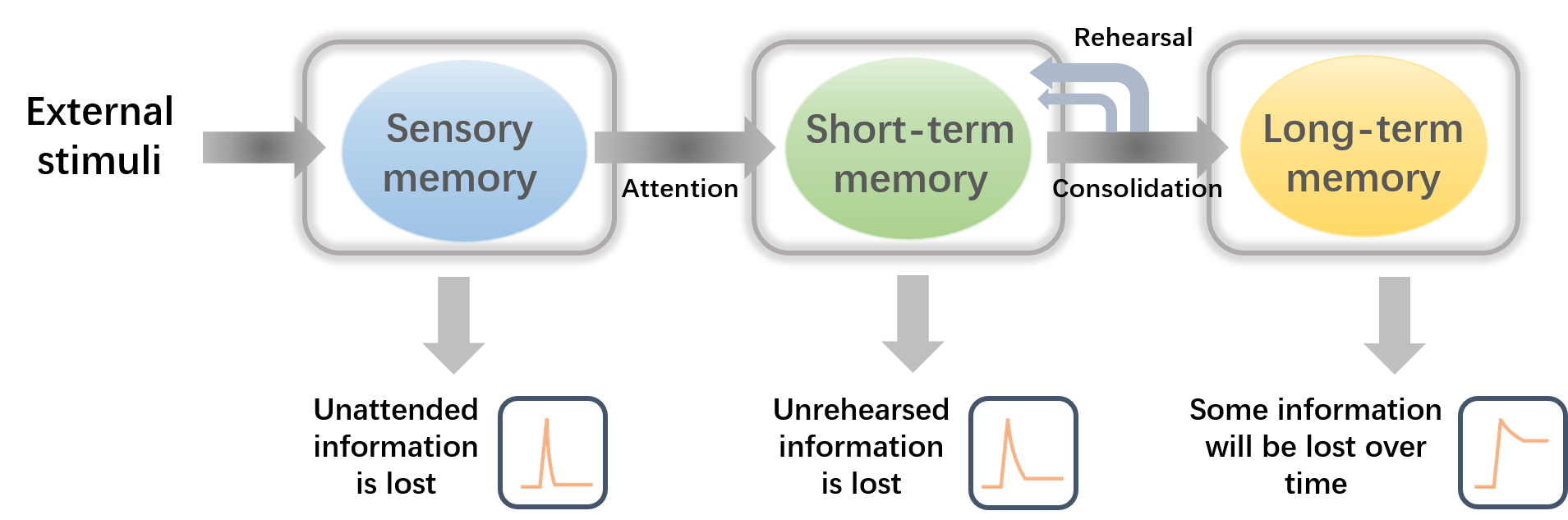


**Figure S6.** Schematic diagram of the memory consolidation process in the human brain.


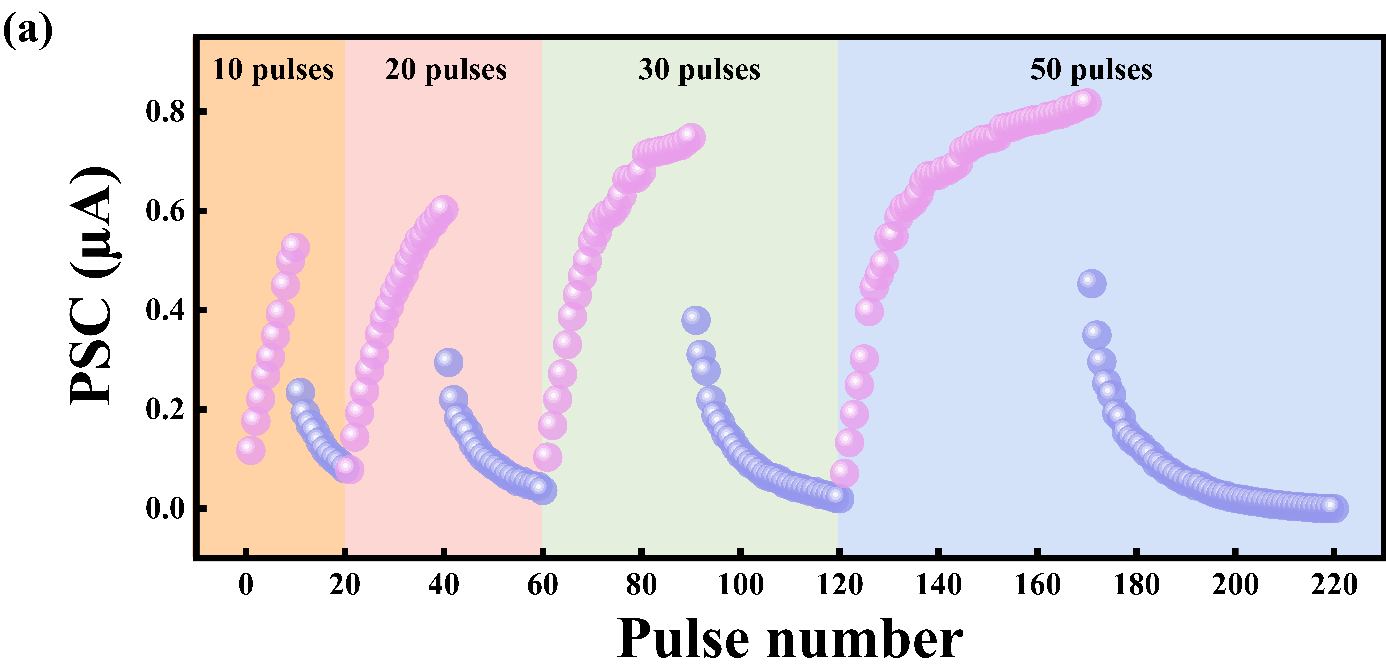


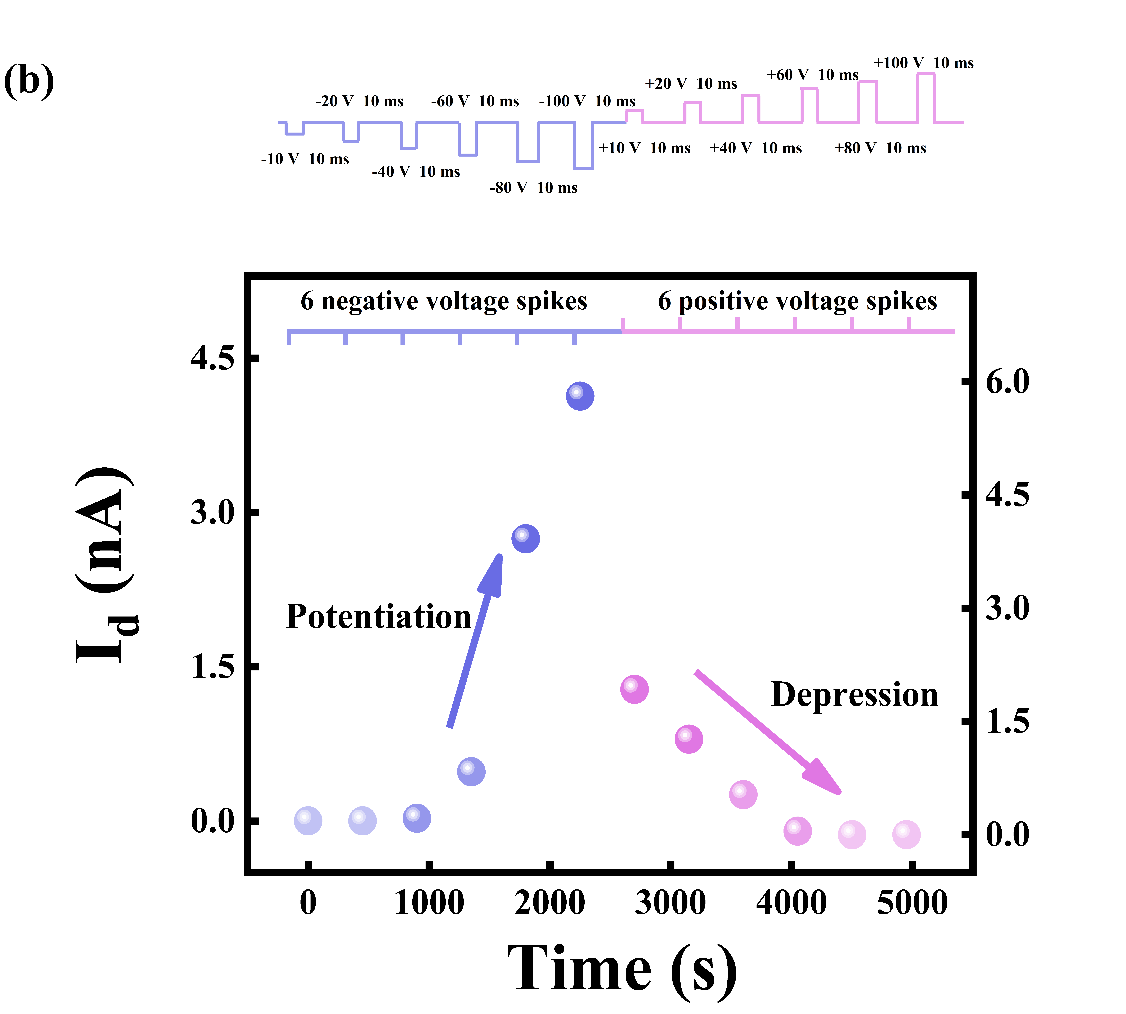


**Figure S7.** **(a)** Performance of potentiation-depression with consecutive 4 sets of different electrical pulse numbers. **(b)** Different amplitudes of 6 negative V_gs_ are applied continuously in order to induce pulse potentiation, next to, different amplitudes of 6 positive V_gs_ are applied continuously for inducing pulse depression.


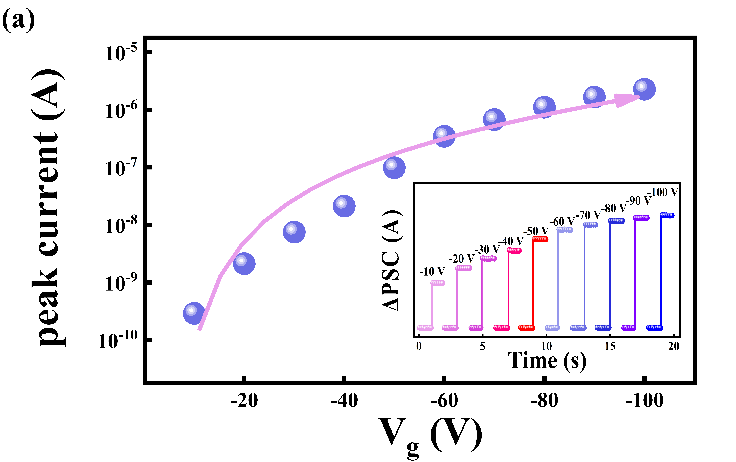

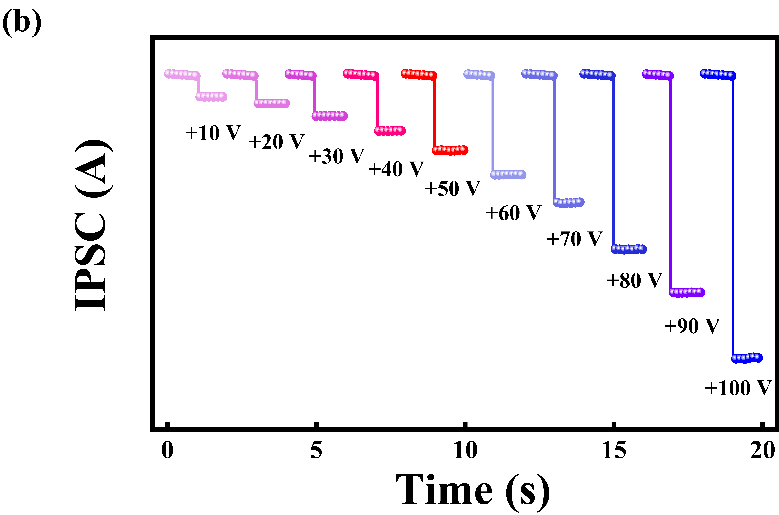


**Figure S8.** **(a)** The synaptic potentiation and depression under the modulation of 10/20/30/50 consecutive negative (-50 V, 10 ms duration) and then 10/20/30/50 consecutive positive (50 V, 10 ms duration) gate pulses. **(b)** The synaptic potentiation and depression triggered by six successively decreasing negative V_g_ pulses (from -10 to -100 V, 10 ms duration ) and then six successively increasing positive V_g_ pulses (from 10 to 100 V, 10 ms duration ).


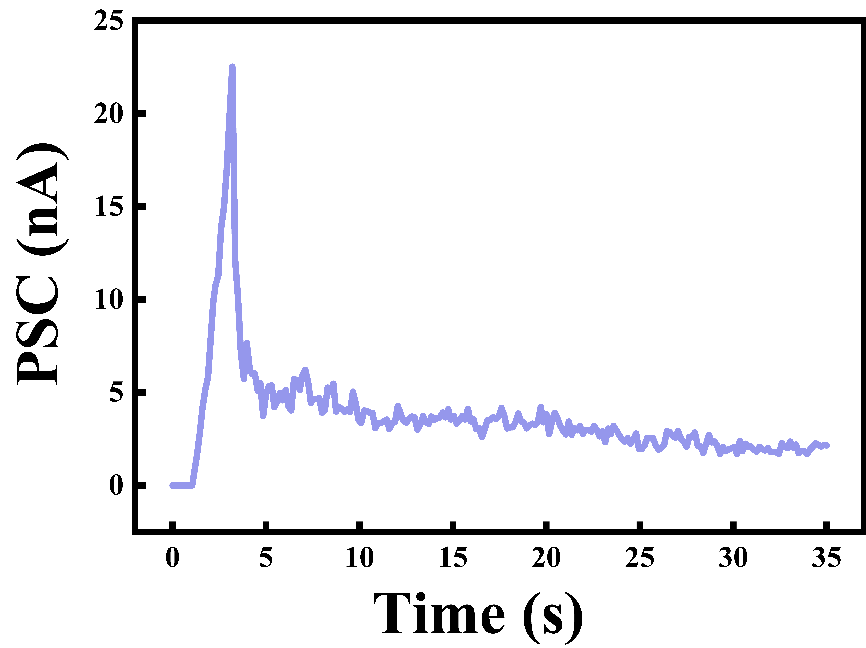


**Figure S9.** The time-resolved retention performance of a bare MoS_2_ transistors after removal of light pulses.

**
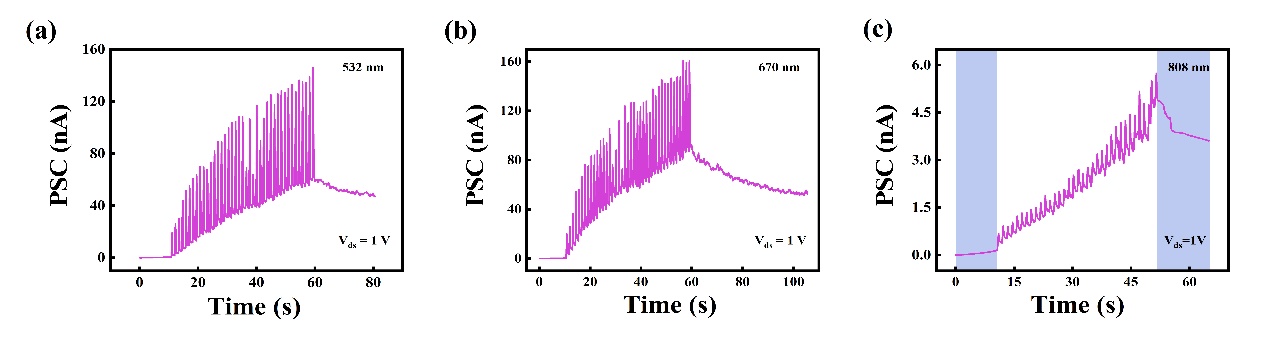
**

**Figure 10.** The PSC triggered by a series of light pulses with the different wavelengths of 532 nm, 670 nm and 808 nm.


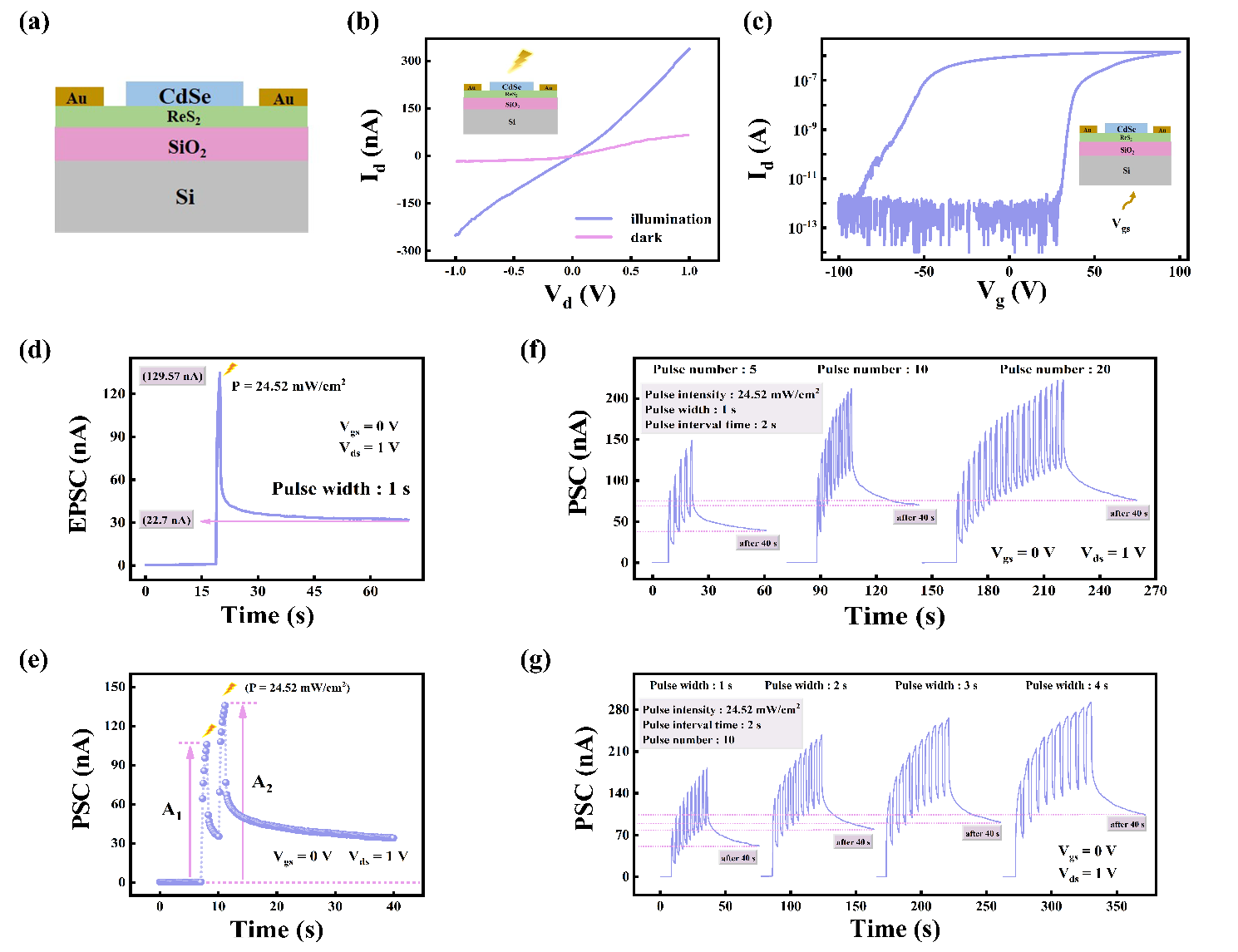


**Figure S11. Design and characteristics of the artificial optoelectronic synaptic device based on the CdSe/ReS_2_ van der Waals heterojunction. (a)** Schematic diagram of the fabricated device. **(b)** The I_ds_-V_ds_ characteristics of the device measured under dark and illumination. **(c)** Typical dual-sweeping transfer curves of the device. **(d)** The transient EPSC response triggered by pre-synaptic light spike (wavelength: 405 nm) with the pulse duration of 1 s. **(e)** EPSC of the CdSe/ReS_2_ transistor excited by two adjacent laser pulses (wavelength: 405 nm, pulse width: 1 s) with a time interval (Δt = 2 s). **(f)** The EPSC response under different numbers of light pulses (the reading voltage is fixed at 1 V). **(g)** The EPSCs triggered by laser pulse trains with different pulse width durations.

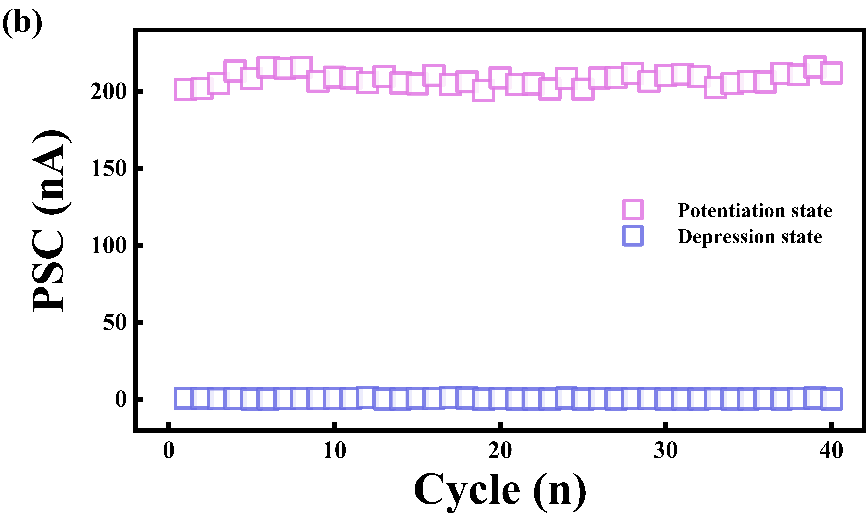

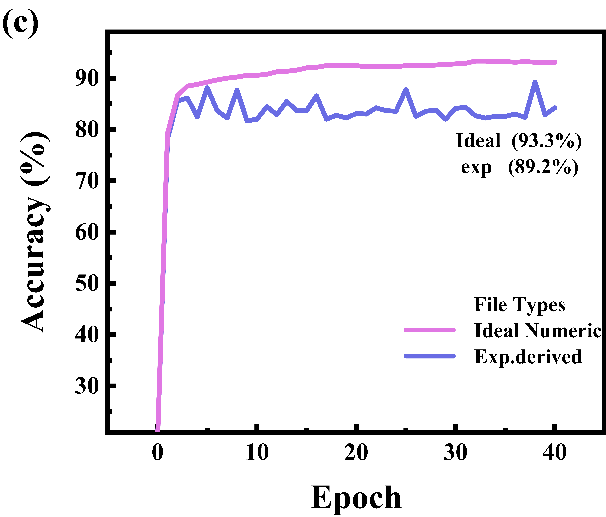


**Figure S12.** (a) The 40 cycles of potentiation/depression curves based on CdSe/MoS_2_ synaptic Transistor. (b) Switching characteristic of the transistor. Data were extracted from the 40 cycles of LTP/LTD curves. (c) The simulated recognition rate as function of training epochs for file type datasets based on the ideal device and our device for the file types.

1. * Corresponding author.

   *E-mail address*: xiacongxin@htu.edu.cn [↑](#footnote-ref-0)
